# The use of traditional medicine in maternity care among African women in Africa and the diaspora: a systematic review

**DOI:** 10.1186/s12906-017-1886-x

**Published:** 2017-08-02

**Authors:** Zewdneh Shewamene, Tinashe Dune, Caroline A. Smith

**Affiliations:** 1 NICM, Western Sydney University, Penrith, NSW 2751 Australia; 2 School of Science and Health, Western Sydney University, Penrith, NSW 2751 Australia; 3 Translational Health Research Institute, Western Sydney University, Penrith, NSW 2751 Australia; 4 Department of Pharmacology, College of Medicine and Health Sciences, University of Gondar, 196 Gondar, Ethiopia

**Keywords:** Traditional medicine, Women, Maternal health, Africa, Diaspora

## Abstract

**Background:**

There is a paucity of literature describing traditional health practices and beliefs of African women. The purpose of this study was to undertake a systematic review of the use of traditional medicine (TM) to address maternal and reproductive health complaints and wellbeing by African women in Africa and the diaspora.

**Method:**

A literature search of published articles, grey literature and unpublished studies was conducted using eight medical and social science databases (CINAHL, EMBASE, Infomit, Ovid Medline, ProQuest, PsychINFO, PubMed and SCOPUS) from the inception of each database until 31 December 2016. Critical appraisal was conducted using a quality assessment tool (QAT).

**Result:**

A total of 20 studies conducted in 12 African countries representing 11,858 women were included. No literature was found on African women in the diaspora related to maternal use of TM or complementary and alternative medicine (CAM). The prevalence of TM use among the African women was as high as 80%. The most common TM used was herbal medicine for reasons related to treatment of pregnancy related symptoms. Frequent TM users were pregnant women with no formal education, low income, and living far from public health facilities. Lack of access to the mainstream maternity care was the major determining factor for use of TM.

**Conclusion:**

TM is widely used by African women for maternal and reproductive health issues due to lack of access to the mainstream maternity care. Further research is required to examine the various types of traditional and cultural health practices (other than herbal medicine), the beliefs towards TM, and the health seeking behaviors of African women in Africa and the diaspora.

**Electronic supplementary material:**

The online version of this article (doi:10.1186/s12906-017-1886-x) contains supplementary material, which is available to authorized users.

## Background

Depending on the cultural or ethnic groups engaging with traditional health practices, the term traditional medicine (TM) or complementary and alternative medicine (CAM) are used, albeit interchangeably. The label CAM is commonly used in studies from Western countries whereas TM is used for developing regions (such as Africa) in which Western medicine is not the predominant health care system.

*The World Medicines Situation 2011 report* estimates that between 70 and 95% of the population in developing countries use TM [1]. In Africa, more than 80% of the population use TM [2]. For the majority, TM is the only accessible primary health care option particularly for the rural African communities [3], and continued use of TM in Africa is likely due to limited accessibility, availability and affordability of modern medicine. In addition, given that TM has a significantly longer history than Western medicine in Africa, there is a deep rooted cultural trust towards TM and traditional medicine practitioners among many Africans [4, 5]. For example, rural African women usually prefer traditional health practitioners such as traditional birth attendants to biomedical health care professionals [6–8].

Use of traditional and complementary medicines for maternity related health complaints is common [9–11]. Although international estimates vary considerably, there appears to be increasing CAM use in maternity with research from many regions showing that up to 87% of women are using some form of traditional and complementary therapies, with more conservative estimates ranging between 20 and 60% [10, 12–14]. Women in Western Societies use CAM for various conditions including (but not limited to) the treatment of premenstrual tension [15], pregnancy related problems [16], back pain [17], infertility [18], postmenopausal symptoms [19], for induction of labor [20].

In most parts of Africa, cultural and traditional health practices play a significant role in maternal health care [7]. In rural Africa, communities tend to adhere to the traditional belief that pregnancy and delivery is the province of traditional birth attendants [21]. Hence, African women perceive traditional healers as primary health care workers [6]. Currently there is a paucity of literature describing the traditional health practices of African women to enhance fertility, promote healthy pregnancy, ensure a normal birth, and promote and maintain health during the postnatal period.

The purpose of this article was to undertake a systematic review describing patterns of TM use for various maternal and reproductive health complaints among women in Africa and the diaspora. More specifically, the review aimed at describing the prevalence of TM use in relation to maternal and reproductive health care, reasons and/or motivators for TM use, common types of maternal and reproductive health complaints treated by TM, types of TMs used, views/perceptions and characteristics of TM users.

In this review, TM refers to “the sum total of the knowledge, skills and practices based on the theories, beliefs and experiences indigenous to different cultures, whether explicable or not, used in the maintenance of health, as well as in the prevention, diagnosis, improvement or treatment of physical and mental illnesses” as defined by the World Health Organization (WHO) [22].

## Methods

### Search strategy

The search included the following eight databases: CINAHL, EMBASE, Infomit, Ovid Medline, ProQuest, PsychINFO, PubMed and SCOPUS and was conducted from the inception of each database until 31 December 2016. The search terms employed were the same for all databases. A detailed search strategy was developed by author (ZS) with input from authors (TD) and (CS). The search strategy combined terms for: (i) women or females, (ii) African or African-born migrants, (iii) traditional medicine, and (iv) maternal health conditions. All possible synonyms of these terms were listed and combined using Boolean operators (see Additional file 1 for more details). The reference lists of all included articles were also checked for other relevant studies.

### Inclusion and exclusion criteria

Studies were included if they reported use of TM by African women or African-born migrant women for reasons related to the preparation for pregnancy, promoting fertility, treating pregnancy related symptoms, maintenance of general wellbeing during pregnancy, inducing or assisting labour, terminating pregnancy (abortion), and enhancing milk secretion or postnatal wellbeing. Studies were also included if describing the views, attitudes or beliefs of women towards TM. The search strategy included primary research (quantitative, qualitative and mixed methods), grey literature and unpublished reports.

Studies were excluded if they focused on women’s use of TM for general purposes and other conditions which were not directly related to maternal health care (e.g. postmenopausal symptoms, breast cancer and prevention of mother to child transmission of HIV). Studies that reported combined use of TM and pharmaceuticals were excluded if the data on TM could not be separated. Ethno-botanical surveys were also excluded.

### Study selection and data extraction

Author ZS conducted the search from November to December 2016. A step-by-step review strategy was implemented to identify all relevant studies. Studies retrieved by the search were assessed first by title and then by abstracts by author ZS. This was followed by reading full texts to identify the final studies for inclusion. Data was extracted according to a predefined reference by all authors (ZS, TD and CS) with disagreement resolved through discussion. The data extracted covered the country of studies, participants’ demographics, prevalence of TM use, details of TMs used, characteristics of TM users, maternal conditions treated by TM, reasons of use, source of information, disclosure of TM use to health professionals, and the method of data collection. All search results were imported into Endnote, a bibliographic management software system and analysed.

### Quality assessment

There is no agreed set of methods for assessing the quality of observational studies describing CAM use [23–25]. Bishop and colleagues recently developed a Quality Assessment Tool (QAT) for a systematic review of the prevalence of complementary medicine use in pediatric cancer [23]. The QAT has also been modified by Grant and colleagues for a systematic review on use of CAM by people with cardiovascular diseases [25]. We have used the modified version of the QAT to undertake quality appraisal of the 18 quantitative studies (two qualitative studies were excluded from appraisal as QAT is not designed to assess quality of such studies). We were also unable to carry out a separate quality analysis for these articles because the number of studies was too small to allow us to draw firm conclusions. Author ZS and CS assessed the quality of studies with disagreements resolved by discussion.

### Reporting and data analysis

A narrative synthesis of studies was undertaken. Data such as prevalence rates were analysed and grouped together for comparison between studies and/or countries. Quality assessment scores were calculated.

## Results

### Study selection and characteristics

**Fig. 1:**
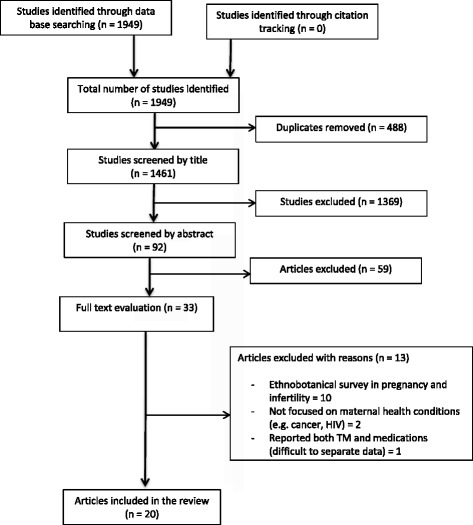
PRISMA flow chart of included and excluded studies

The database search identified 1949 potential references, from which 488 duplicates were removed. A total of 92 references were reviewed by abstract and 59 were not directly related to the aim of the review. The remaining 33 articles were examined by full text and a total of 20 articles were included [7, 8, 26–43]. Thirteen articles which examined an ethno-botanical survey of herbs used in pregnancy, focused on use of both TM and pharmaceuticals, reported the use of TM for treatment of HIV and cancer were excluded (Fig. 1).

Of the 20 studies included, four were conducted in Nigeria [28, 30, 40, 41], three in South Africa [8, 33, 43], and two each in Ethiopia [27, 32], Uganda [31, 37] and Tanzania [39, 42]. The remaining seven studies were conducted in Zimbabwe [35], Zambia [26], Mali [36], Lesotho [34], Kenya [7], Morocco [29], and Egypt [38]. No study was found related to maternal use of TM or CAM among African women of the diaspora.

Studies were published between 1985 and 2016, from which 13 (65%) were published between 2014 and 2016. Of the selected articles, 17 studies were quantitative while two were qualitative. One study utilised a mixed research method which included a structured questionnaire survey with pregnant women and focus group discussions involving TM providers. From this study, the data that concerned only the women was extracted in keeping with the aim of this review.

### Sample and study setting

Twenty studies investigated the use and/or the perception of TM for various maternal health conditions among 11, 858 African women. The sample size of the quantitative studies ranged from 72 to 5686 participants. Five studies included a sample of 500 or more participants [26, 30, 33, 40, 41]. Thirteen (72.2%) studies included pregnant women who were currently attending health facilities [26–28, 30, 32–38, 41, 42]. Five studies focused on surveying women who were mothers or had been pregnant in the past 2–5 years [7, 8, 29, 40, 43] preceding their data collection. Other studies sampled nursing mothers [28], women attending a fertility clinic [31], and women who underwent unsafe abortion [39]. Most studies reported on women of childbearing age (18–45 years).

### Study quality

As shown in Table 1, the modified QAT included four major assessment criteria: i) study methodology, ii) sampling, iii) participant characteristics, and iv) TM use. A total of 15 specific quality assessment criteria were weighted according to their relative importance as described by Bishop and colleagues [23]. Three items scored a maximum of 2 points, 8 items scored 1 point, and 4 items scored 0.5 points. The maximum score was 16.

**Table 1:** Summary of the quality of studies

Quality assessment items		Brief description	Points awarded	Percentage/frequency of studies % (n)	Reference
Study methods					
Recall bias	Low risk	Prospective data collection	2	-	-
	Some risk	Retrospective data collection within previous 12 months	1	33.3 (6)	[7, 26–28, 31, 42]
	High risk	Retrospective data collection not within previous 12 months	0	66.7 (12)	[29, 30, 32–41]
Piloted questionnaire (or interview schedule)		Any pilot, feasibility, pretest, or previous use of study materials	1	61.1 (11)	[28, 30–32, 34, 35, 37, 38, 40–42]
Address potential sources of bias		Report efforts to address nonresponsive bias or information bias	1	16.7 (3)	[28, 30, 35]
Adjust for potential confounders		Any adjustment of confounders in analyses of variables associated with TM use	1	27.8 (5)	[28, 31, 32, 37, 40]
Sampling					
Response rate		Where response rate = (number of participants in the study/number of people invited to take part) × 100	1	44.4 (8)	[7, 26, 29–32, 36, 40]
Representative sampling strategy		Any attempt to achieve a sample of participants that represents the larger population from which they were drawn (but cannot be a single center sample)	1	16.7 (3)	[30, 35, 42]
Participant characteristics					
Specific diagnosis		Report participants’ diagnoses	1	22.2 (4)	[26, 29, 31, 39]
Indicator of socioeconomic status		Report participants’ socioeconomic status	0.5	83.3 (15)	[7, 26–30, 32–38, 40, 41]
Age		Report participants’ ages	0.5	88.9 (16)	[7, 26–38, 41, 42]
Ethnicity		Report participants’ ethnicity	0.5	27.8 (5)	[26–28, 35, 42]
Gender		Report participants’ gender	0.5	100 (18)	[7, 26–42]
TM use					
TM definition		Information about the definition of TM/a list of TM modalities provided to participants	2	27.8 (5)	[7, 30, 34, 35, 41]
Use of TM modalities assessed		Report the prevalence of use of specific TM modalities	1	88.9 (16)	[7, 27–39, 41, 42]
Frequency/duration of TM uses		Report how often or for what duration the TM were/are used by study participants	1	11.1 (3)	[35, 36, 42]
Reasons for TM use		Report the reasons for the use of TM by study participants	2	61.1 (11)	[27, 29–31, 33, 34, 36–39, 42]

Study quality varied significantly between studies with QAT percentage scores ranging from 25% to 59.4%. Fifteen studies attained less than 50% of the maximum score [7, 26–29, 32–34, 36–42] (Table 2). All quantitative studies used retrospective data collection methods in which there was a potential for risk of recall bias. Only five studies used a retrospective data collection within the 12 months to minimise the risk of recall bias [7, 26–28, 31]. Eleven studies (61.1%) collected data with a piloted questionnaire, while five (27.8%) studies adjusted for potential confounders in their analysis. Seven studies reported a response rate which ranged between 74% and 100%. Only three studies recruited a multicenter sample in an attempt to achieve a representative sample of participants to the larger population from which they were drawn. Fifteen studies collected socio-economic data and 16 studies reported the age of participants. Only five studies reported the ethnicity of the study participants, most studies were from regions or countries of homogenous populations.

**Table 2:** Quality assessment of individual studies

Author/ year of publication	Study methods (5 points)				Sampling (2 points)		Participant characteristics (3 points)					TM use (6 points)				Total points awarded (Max = 16)
	Recall bias (2 pts) (2 = low risk if data collection was prospective; 1 = some risk if data collection is retrospective within previous 12 months; 0 = high risk)	Piloted questionnaire or interview schedule (1 pt)	Address potential source of bias (1 pt)	Adjust for potential confounders (1 pt)	Response rate (1 pt)	Representative sampling (1 pt)	Specific diagnosis (1 pt)	Indicator of socioeconomic status (0.5 pt)	Age (0.5 pt)	Ethnicity (0.5 pt)	Gender (0.5 pt)	TM definition (2 pts)	Use of TM modalities assessed (1 pt)	Frequency/duration of TM use (1 pt)	Reasons for TM use (2 pts)	
Banda et al., 2007 [26]	1	0	0	0	1	0	1	0.5	0.5	0.5	0.5	0	0	0	0	5 (31.3%)
Bayisa et al., 2014 [27]	1	0	0	0	0	0	0	0.5	0.5	0.5	0.5	0	1	0	2	6 (37.5%)
Duru et al., 2016 [28]	1	1	1	1	0	0	0	0.5	0.5	0.5	0.5	0	1	0	0	7 (43.8%)
Elkhoudri et al., 2016 [29]	0	0	0	0	1	0	1	0.5	0.5	0	0.5	0	1	0	2	6.5 (40.6%)
Fakeye et al.,2009 [30]	0	1	1	0	1	1	0	0.5	0.5	0	0.5	1^a^	1	0	2	9.5 (59.4.6%)
Kaadaaga et al., 2014 [31]	1	1	0	1	1	0	1	0	0.5	0	0.5	0	1	0	2	9 (56.3%)
Lalego et al., 2016 [32]	0	1	0	1	1	0	0	0.5	0.5	0	0.5	0	1	0	0	5.5 (34.4%)
Mabina et al., 1997 [33]	0	0	0	0	0	0	0	0.5	0.5	0	0.5	0	1	0	2	4.5 (28.1%)
Mbura et al., 1985 [42]	1	1	0	0	0	1	0	0	0.5	0.5	0.5	0	1	0	2	7.5 (46.9%)
Mothupi and Carol 2014 [7]	1	0	0	0	1	0	0	0.5	0.5	0	0.5	1^a^	1	0	0	5.5 (34.4%)
Mugomeri et al., 2015 [34]	0	1	0	0	0	0	0	0.5	0.5	0	0.5	2	1	0	2	7.5 (46.9%)
Mureye et al., 2012 [35]	0	1	1	0	0	1	0	0.5	0.5	0.5	0.5	1^a^	1	1	0	8 (50%)
Nergard et al., 2015 [36]	0	0	0	0	1	0	0	0.5	0.5	0	0.5	0	1	1	2	6.5 (40.6%)
Nyeko et al., 2016 [37]	0	1	0	1	0	0	0	0.5	0.5	0	0.5	0	1	0	2	6.5 (40.6%)
Orief et al., 2014 [38]	0	1	0	0	0	0	0	0.5	0.5	0	0.5	0	1	0	2	5.5 (34.4%)
Rasch et al., 2014 [39]	0	0	0	0	0	0	1	0	0	0	0.5	0	1	0	2	4.5 (28.1%)
Sarmiento et al., 2016 [40]	0	1	0	1	1	0	0	0.5	0	0	0.5	0	0	0	0	4 (25%)
Tamuno et al., 2011 [41]	0	1	0	0	0	0	0	0.5	0.5	0	0.5	2	1	0	0	5.5 (34.4%)

^a^Studies that explained the definition of TM or provided list of TM modalities to their participants

Three studies reported they provided a definition of TM to study participants, although the definition was not stated in the manuscripts (8, 12, 14). Two articles provided an operational definition of TM for their study (13, 20). While all studies reported the prevalence of use of TM, only three studies reported the frequency or duration TM use.

### Prevalence of TM use in maternity care

**Fig. 2:**
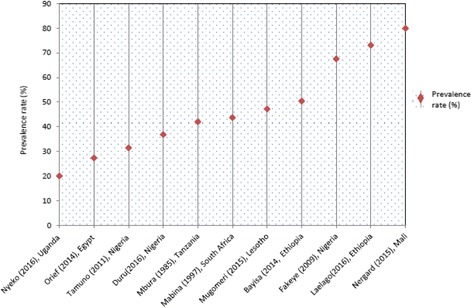
Prevalence of herbal medicine use among pregnant women in Africa

In 18 quantitative studies, prevalence of TM use for maternal and reproductive health issues ranged from 12% [7] to 79.9% [36]. In 15 studies, the TM modality investigated was limited to herbal medicine only, 11 of these studies focused on the use of herbal medicine during pregnancy [27, 28, 30, 32–34, 36–38, 41, 42]. The prevalence of herbal medicine use among pregnant women in these 11 studies ranged from 20% [37] to 79.9% [36] (Fig. 2). A study in rural Tanzania reported a 43% prevalence rate of medicinal herb usage to induce abortion [39]. Another study from Uganda that examined herbal medicine use among women attending the infertility clinic reported a 76.2% prevalence rate.

### Types of TM used

Herbal medicines were mostly investigated (Table 3). Ten studies reported the specific types of herbal preparations commonly used by respondents [8, 27–29, 32, 36, 38, 39, 41, 43]. Some of these studies used names of herbs in local languages while others used either English or Latin names of specific medicinal herbs. Frequently reported herbal medicines included ginger, garlic, aniseed, fenugreek, green tea, peppermint, eucalyptus, rue, garden cress, madder, cinnamon, bitter leaf, palm kernel, bitter kola, neem leaves, and jute leaves. Some of the medicinal herbs reported by their scientific names were *Bidens pilosa, Commelina africana, Desmodium barbatum, Manihot esculenta, Ocimum suave and Sphaerogyne latifolia., Obetia radula, Rubia cordifolia, and Triumfetta microphylla, Lippia Chevalieri, Combretum micranthum, Parkia biglobosa, Peris heterophylla, Stylosanthes erecta, Ximenia Americana, Mitragyana inermis,and Combretum glutinosum* [36, 39]. In one study, the use of different types of TM and other substances by pregnant women was reported [35]. These included holy water, soil from a burrowing mole, pouzolzia mixta, elephant dung, manual exercises, cocktails of unknown herbs, *Cannabis stivum*, castor oil, rooibos tea, and hot water/steam baths.

**Table 3:** Characteristics of studies

Author	Participants’ country of origin	Sample size	Target groups	Prevalence of TM use	Specific types of TMs used	Characteristics of users	Maternal conditions treated by TM/ reasons of use	Source of information or providers	Disclosure of TM use to health care providers	Study design/data collection method
Banda et al., 2007 [26]	Zambia	1128	Pregnant women	21%	NR	- Users are not different from non- users in terms of age, education, ethnicity or income - women who knew anyone who had used TM during pregnancy were more likely to use TM - Women who thought that the use of TM may hurt their baby were less likely to use TM - Women who reported accessing traditional medical care were only half as likely to adhere to HIV drugs	NR	NR	64% of users did not want to share their use of TM to health care providers	Quantitative/Interviewer administered questionnaire
Bayisa et al., 2014 [27]	Ethiopia	250	Pregnant women	50.4%	Herbal medicine (garlic, ginger, eucalypt, ruta rue)	- Age, educational status, marriage, ethnicity and source of information were not associated with TM use - About 70% of users were pregnant women on their first trimester	For treatment of nausea, morning sickness, vomiting, cough, nutritional deficiency	Neighbors, family, health professionals, traditional healers	NR	Quantitative/ semi-structured questionnaire
Duru et al., 2016 [28]	Nigeria	500	Pregnant women and nursing mothers	36.8%	Herbal medicine (bitter leaf, palm kernel, bitter kola, neem leaves, garlic, jute leaves, ginger	- Pregnant women aged 20–30 years were frequent users (41%) - Married women were eight times more likely to use TM than unmarried women - Women with no formal education reported the highest use (85.7%) compared to tertiary education achievers (18.8%) - Better income favored use of TM - Gestational period, parity, ethnicity and occupation did not impacted on the use of TM	NR	NR	NR	Quantitative/ semi-structured interview administered questionnaire
Elkhoudri et al., 2016 [29]	Morocco	181	Mothers who gave birth in the last 5 years preceding the study	42%	Herbal medicine (vervain, cresson, madder, fenugreek, cinnamon, ginger)	- Illiterate women have used TM more frequently - Multiparous women were more likely to use TM than first time mothers	To get back in shape after delivery, facilitate child birth, vomiting, increase breast milk secretion	NR	NR	Quantitative/ interviewer administered questionnaire
Fakeye et al.,2009 [30]	Nigeria	595	Pregnant women	67.5	Herbal medicine (detail is not reported)	- Age, geographical zones and educational status were strongly associated with TM use (detail description of age category and education level were not reported)	Users perceived better effectiveness to TM than conventional medicine, cultural beliefs to TM, better accessibility, lower cost and other reasons were reported	Local herb sellers, herbalists	56.6% of participants did not support combining with herbs with medications	Quantitative/ structured questionnaire
Kaadaaga et al., 2014 [31]	Uganda	260	Women with fertilization problem	76.2	Herbal medicine (detail is not reported)	- Married women with infertility problem were more likely to use TM - women who did not conceived before were more likely to use TM Women with infertility for less than 3 years were more likely to use TM	Treatment of infertility	NR	63.8% of users did not disclose TM use to their physicians	Quantitative/interviewer administered structured questionnaire
Lalego et al., 2016 [32]	Ethipiopia	363	Pregnant women	73.1	Herbal medicine (ginger, garlic, eucalyptus, ruta rue, ocimumlamifolium, garden cress	- being on first trimester, less education and having less knowledge about TM favored use of TM	Management of nausea, vomiting, abdominal pain, cold, fever	Parents/relatives, neighbor, herbalists	NR	Quantitative/ interviewer administered structured questionnaire
Mabina et al., 1997 [33]	South Africa	577	Pregnant women	43.7	Herbal medicine	- Those having knowledge about herbal medicine and on second trimester were frequent users of TM	NR	Parents, relatives, TBA, herbalist, friends	NR	Quantitative/ questionnaire
Mbura et al., 1985 [42]	Tanzania		Pregnant women	42%	Herbal medicine	- Prevalence of TM use among pregnant women from the rural and urban areas has no difference Pregnant women on their first trimester were frequent users - Muslims were frequent users of TM compared to Christians	To treat pregnancy related symptoms, to assist labor	NR	NR	Quantitative/ interview administered questionnaire
Mothupi and Carol 2014 [7]	Kenya	333	Mothers who gave birth in the past 9 months before the study	12%	Herbal medicine (detail was not provided)	- Women with no formal education were more likely to use TM - Women who live far from health facilities (>10 km) were frequent users	To treat swollen feet, back pain, digestive problems. High cost, inaccessibility and distance of health facilities resort respondents to TM use	Family, friends, open markets, herbal clinics	Only 12.5% of user disclosed use of TM to their doctors. About 51% of users reported use of combined herbs with pharmaceutical drugs	Quantitative/ interviewer administered questionnaire
Mugomeri et al., 2015 [34]	Lesotho	72	Pregnant women	47.2	Herbal medicine (detail was not reported)	- 50% of users were on the second trimester - Women’s age, marital status, literacy and parity were not associated with use of TM	Prevention of abortion, prevention of placenta praevia, promotion of fetal growth, edema, spiritual cleansing and relief of pain	Grandmothers, mothers-in-law, TH, TBA	NR	Quantitative/ semi-structured questionnaire
Mureye et al., 2012 [35]	Zimbabwe	248	Pregnant women	52%	TM (holy water, soil burrowed by moles, elephant dung, cocktails of unknown herbs, lubricants and others	- Being in the age range of 20–25, nulliparity and nulligravidity predicted frequent use of TM - Most users were on their third trimester	To prevent perineal tearing, placenta retention, breech presentation, postpartum hemorrhage, prolonged labor and preeclampsia	NR	NR	Quantitative/ interviewer administered questionnaire
Nergard et al., 2015 [36]	Mali	209	Pregnant women and mothers	79.9%	Herbal medicine (Lippia chevalieri, combretum micranthum and others)	- Socio-demographic characteristics were not associated with use of herbal medicines - Frequent use of herbal medicines was reported during the first trimester	For general wellbeing, as dietary supplements, to treat edema, urinary tract infection, and tiredness	NR	Pregnant women used herbal preparation without any supervision from care providers	Quantitative/ interviewer administered questionnaire
Nyeko et al., 2016 [37]	Uganda	383	Pregnant women	20%	Herbal medicine (detail wan not reported)	- Women who used herbal medicine in the past were eight times more likely to use during the current pregnancy - Distance more than 5 km to health facilities was associated with increased herbal medicine use	To treat waist pain, fever, nausea and vomiting. For induction of labor and difficulty in accessing health facilities.	NR	90% of users did not disclose to their health care providers	Mixed method / questionnaire survey and FGDs
Orief et al., 2014 [38]	Egypt	300	Pregnant women	27.3	Herbal medicine (Aniseed, fenugreek, ginger, garlic, green tea and peppermint)	- Statistically significant difference was found regarding the age, gravidity, parity and BMI among the pregnant women who used herbal medicines (details were not reported)	To treat abdominal colic during pregnancy, nausea and vomiting and headache	Friends, family, physician	NR	Quantitative/ questionnaire survey
Rasch et al., 2014 [39]	Tanzania	125	Women who had unsafe abortion	43%	Herbal medicine (*Bidens pilosa*, rubia cordifolia, ocimum suave and others)	- 22% of users ingested medicinal plants orally to induce abortion - 13% of users inserted plant specimens virginally to induce abortion - socio-demographic characteristics of users were not reported	To induce abortion	NR	NR	Quantitative/ interviewer administered questionnaire
Sarmiento et al., 2016 [40]	Nigeria	5686	Pregnant women in the past 2 years	24.1%	NR	- Socioeconomic factors were not associated with use of TM	To assist childbirth	NR	NR	Quantitative/ interviewer administered questionnaire
Tamuno et al., 2011 [41]	Nigeria	500	Pregnant women	31.4%	Herbal medicine (ginger, garlic	- Women with no formal education were more likely to use TM - Low socio-economic status was significantly associated with TM use	NR	NR	Over 40% of women reported combined use of herbs and drugs	Quantitative/ self-administered questionnaire
Naidu 2014 [43]	South Africa	21	women who were either pregnant or women who had had children	NA	*Isihlambezo (*Herbal decoction used by many Zulu women in South Africa as a preventative health tonic during pregnancy)	Women’s have a strong cultural belief to *Isihlambezo* to prevent health problems during pregnancy	NA	NA	NR	Qualitative/ interview
Kooi and Theobald 2006 [8]	South Africa	27			*kgaba (*contains different herbal medicines to prevent physical problems and the perceived harm that evil spirits can cause during pregnancy)	The use of *kgaba* as perceived by the women is an important component in the experience of pregnancy and labour	NA	NA	communication about the use of *kgaba* between pregnant women and health staff was poor	Qualitative/interview

NR = not reported; NA = not applicable

### Determinants of TM use

Lack of access to western medicine was reported to be the major determining factor that influenced women to use TM [7, 30, 36, 42]. Other determinants included user’s belief that TM was more effective than western medicine, as well as better accessibility and lower cost of TM.

A study from Nigeria reported significantly increased use of TM among pregnant women aged 20–30 years, with no formal education, a good income (those earning above 250 dollars monthly) and who were married [28]. In this study, gestational age, parity, ethnicity and occupation did not affect the use of TM. Another study identified that married women, women within the first three years of infertility diagnosis, and those never conceived were more likely to use TM [31]. The authors also noted that the majority of the participants sought biomedical care for infertility after three years of being unable to achieve a pregnancy. In most of the African culture, the main purpose of marriage is procreation, and if a woman fails to conceive soon after marriage, they seek help from traditional healers [31, 44].

A study form Ethiopia reported that during the first trimester of pregnancy, being less educated and having less knowledge about TM were associated with higher use of TM [32]. A Ugandan study found that women who used herbal medicine in the past were eight times more likely to use during the current pregnancy [37]. In two studies, a long distance to health facilities was found to be associated with increased herbal medicine usage during pregnancy [7, 37]. In one study which investigated the use of TM during childbirth, socioeconomic factors were not significantly associated with use of TM [40]. Two studies found multiparous women were more likely to use TM than first time mothers [29, 34]. In a Zambian study, users were not different from non-users in terms of age, education, ethnicity or income [26]. In this study, women who knew someone who had used TM during pregnancy were more likely to use TM, and women who thought that the use of TM may hurt their baby were less likely to use TM.

### Reasons for TM use

Fourteen studies reported reasons for TM use [7, 27, 29–32, 34–40, 42]. Some of the reasons were related to treating pregnancy related conditions including nausea, vomiting, nutritional deficiency, swollen feet, back pain, digestive problems, fever, cold, abdominal pain, edema, urinary tract infection, tiredness, headache, and waist pain [7, 27, 32, 36, 37] (Table 3). One study reported that TM was frequently used to get back in shape after delivery, facilitate child birth, increase breast milk secretion and reduce pain during pregnancy [29].

Two studies reported that the main reasons for TM use were for prevention of perineal tearing, placenta retention, breech presentation, postpartum hemorrhage, prolonged labor, preeclampsia, abortion and pains [34, 35]. Other reasons for TM use included general wellbeing during pregnancy, promotion of fetal growth, spiritual cleansing, to protect the pregnancy against evil influence, to have a male baby, for induction of labour, assisting childbirth, and as dietary supplements [34–37, 40, 42].

### Views of women towards TM

Two qualitative studies from South Africa reported the constructed meaning, knowledge, and beliefs of pregnant women and mothers towards specific types of traditional health practices. Kooi and Theobland examined the women’s belief about kgaba remedy, a traditional therapy based on a mixture of plants and minerals that can vary among traditional healers and has not been officially documented [8]. Authors noted that Kgaba may contain mixture of up to 18 different medicinal herbs and can be prepared by combining these herbal remedies with ostrich eggshell, baboon urine, mud, and ashes of burnt herbs. They concluded that kgaba was an important component in the experience of pregnancy and labour, mainly to protect pregnant woman from evil and harm, and to induce labour. Similarly, Naidu examined the constructed meaning of isihlambezo, a herbal mixture used by many Zulu women in South Africa as a preventative health tonic during pregnancy [43]. The author reported that pregnant women perceive isihlambezo as a powerful medicine given by God and the ancestors to protect the mother and her unborn baby. The strong belief in the efficacy of isihlambezo was echoed in the understanding of many of the participants.

### Sources of information on TM

The source of information on TM was not addressed in the majority of studies. Four studies identified the sources of information on TM were from families, relatives, friends, traditional birth attendants, and local herb sellers/herbalists [27, 30, 32, 41].

### Concurrent use of TM with prescription drugs and disclosure to clinicians

Three studies reported the concurrent use of TM with prescription drugs [7, 30, 41]. In one study, although respondents used prescribed pharmaceuticals and herbal medicine concomitantly, few users disclosed their use to a healthcare professional [7]. In this study, when use of herbal medicine was disclosed, participants reported that the healthcare providers provided advice about side effects, or discouraged the use. One study found 40% of those using herbal medicine combined herbs with pharmaceutical drugs during pregnancy [41]. In another study, 13.7% of herbal medicine users reported that they support the use of TM combined with prescription medications during pregnancy [30].

Four studies reported data on disclosure of TM use to health care providers [7, 26, 31, 37]. In one study, 64% of users did not want to share their use of TM to clinicians. Reasons given for nondisclosure to physicians about TM use included fear that it might negatively impact their antenatal care [26]. Another study also reported 63.8% of users did not disclose TM use to attending physicians although their reason was not examined [31], whereas another study indicated 89.7% of respondents were not interested in disclosing use of herbal medicine to the healthcare providers [37].

## Discussion

TM use is common for maternal and reproductive health complaints among African women. Our findings of high prevalence of TM use in maternity care is consistent with the prevalence in the general African population, where TM is regarded as the primary health care option for most rural communities [3]. In addition, there is greater access to traditional health practitioners in these communities compared to the availability of Western health care providers [6]. For example, traditional birth attendants and herbalists are easily accessible in most African villages [6, 43].

Notably, the prevalence of TM use in maternity varies significantly (21%–79.9%). This substantial variation could be attributed to the lack of specific and consistent definitions of TM in the reviewed studies. Differences in the study settings (e.g., rural versus urban) which are not reported in most of the reviewed articles may also contribute to the variability of observed findings. Other influencing factors may be variable age ranges and sampling techniques.

This review found that TM has been used for various maternal health issues including the treatment of pregnancy related symptoms [7, 27, 32, 36], induction of labor [29, 34, 35], facilitating breast milk secretion [29], inducing abortion [39], treatment of infertility [31] and to maintain general wellbeing during pregnancy [29, 34–36]. These results are in agreement with studies in Western societies where CAM is used before pregnancy, during pregnancy and labor and extending into the postpartum period [45–48].

Frequent TM users in most of the studies were women with no formal education and low income. This may be related to the fact that many women living in rural villages in Africa have less opportunity for education and employment [49]. This may in turn limit women’s knowledge about available healthcare options outside of traditional and cultural health practices. Additionally, some African women experience limited autonomy as compared to their male counterparts who may be responsible for making decisions concerning women’s health care choices and wellbeing [50, 51]. African women may also embrace traditional beliefs and practices that influence their use of TM as a maternity health care choice. For instance, they trust the knowledge of traditional birth attendants, and prefer their care and expertise to the treatment that they receive from midwives in hospitals and clinics [52].

Lack of access to maternity health services was a common predictor of TM use in most of the studies. This relationship may partly be explained by limited accessibility, availability, and affordability of Western health care services [4]. Many rural African women also need to travel longer distances to receive modern maternity health care [53]. Although most studies in this review failed to report their study setting (urban versus rural), a study conducted among women living in urban areas, where modern health facilities are available, found lower prevalence of TM use [7]. This may suggest that distance to maternity clinics may lead to increased TM practice by women living in rural areas.

Common sources of information on TM were from the recommendation of family, friends and traditional birth attendants. Studies have shown that advice from family and friends is trusted more compared to other sources [4, 54]. African TM is also rooted within cultures, and the information is handed down between close family members [4, 55]. The role of traditional birth attendants in maternal health care in Africa has been shown to be highly trusted and valued. This is because African women perceived birth attendants to be cultural custodians as demonstrated by their practices [56]. This may contribute to birth attendants place as a primary information source and service providers of TM for many African women.

Some studies reported combined use of prescribed pharmaceuticals and herbal medicine and lack of communication with health professionals [7, 26, 30, 31, 37, 41]. The major reasons for non-disclosure of TM use to clinicians included fear that disclosure might negatively impact their treatment, that it was not asked about by attending physicians and women’s unwillingness to discuss the use of traditional health practices with health professionals. This may occur due to health professionals’ lack of awareness or perception that they have judgmental and dismissive attitudes about the cultural and traditional health practices and needs of TM using women in Africa [7, 31].

The strength of most studies was seen in reporting participants’ age, socio-economic status, and reasons for TM use. However, the overall quality of the studies was low, with only three of the studies scoring 50% or more on the modified QAT. This was mainly because many studies collected data on a retrospective basis which may introduce significant recall bias. In addition, studies failed to adjust confounders in analyses of variables associated with TM use. The lack of a specific operational definition of TM also affected the quality of studies. Studies also failed to use validated surveys and representative samples.

### Strengths and limitations

This is the first comprehensive review to date of studies reporting the use of TM for maternal health among African women, and it increases our understanding of African women’s traditional health practices and highlights an extensive use of TM. A rigorous search strategy was conducted of the English language literature identifying published articles, grey literature and unpublished studies to minimise the potential for publication bias. However, the studies included focused primarily on use of herbal medicine during pregnancy, and therefore limit our understanding about other African traditional and cultural health practices. Lack of studies among the African diaspora also limits the generalizability of this finding to migrant African women.

### Practical implications

Findings of this review may help policy makers as well as conventional and traditional health providers understand the traditional health practices and beliefs of African women. Identification of these traditional practices and the reasons why women choose to use them may support the development of strategies to improve maternal and reproductive health services for African women. It may also lead to better regulation of TM practices and the identification of gaps for future research.

### Implication for future research

Although TM is a primary source of health care for more than 80% of the African population, there is little information regarding the types of African TM, users’ profile, and reasons for and determinants of use. There is also an absence of research examining TM use among African-born migrant women and how their previous cultural health practices and beliefs may influence their health seeking behavior. Research that includes a broader operational definition of TM is needed to fully understand the traditional and cultural health practices and beliefs of African women in Africa and the diaspora. The reasons and determinants for higher reliance on TM among Africa women also need further investigation.

## Conclusions

The results of this systematic review highlight the high level of TM use by African women to address their maternal and reproductive health issues. Studies have primarily focused on TM use during pregnancy, and herbal medicine was the most commonly used TM but also a treatment modality that studies have exclusively examined. Pregnancy related health complaints were the common reason that women sought treatment with TM, while lack of access to mainstream health care was the main driving factor to use TM. The quality of the reviewed studies was generally poor and there was incomplete reporting to address important methodological biases. Studies of the African diaspora women are lacking. Further research is required to examine the various types of traditional and cultural health practices (other than herbal medicine), the beliefs towards TM, and the health seeking behaviors of African women in Africa and the diaspora.

## Additional file

Additional file 1:
**Appendix A.** Search terms and number of records found from each databases. (PDF 362 kb)

## Abbreviations

CAM: Complementary and alternative medicine
NICM: National Institute of Complementary Medicine
QAT: Quality Assessment Tool
TM: Traditional medicine
WHO: World Health Organization
