# Supplementary material for: The use of traditional medicine in maternity care among African women in Africa and the diaspora: a systematic review
Source: BMC Complement Altern Med. 2017 Aug 2;17:382. doi: 10.1186/s12906-017-1886-x (PMC5541739; doi:10.1186/s12906-017-1886-x)
Supplement: Additional file 1: — Appendix A. Search terms and number of records found from each databases. (PDF 362 kb) [file 12906_2017_1886_MOESM1_ESM.pdf]

Appendix A: Search terms and number of records found from each databases

Database	Search string	Number of records
CNAHL	AB (Women OR females OR mothers) AND TX (Africa* OR "African migrant" OR "African-born" OR " African immigrant" OR Algeria OR Angola OR Benin OR Botswana OR "Burkina Faso" OR Burundi OR Cameroon OR "Cape Verde" OR "Central African Republic" OR Chad OR Comoros OR "Ivory Coast" OR Congo OR Djibouti OR Egypt OR "Equatorial Guinea" OR Eritrea OR Ethiopia OR Gabon OR Gambia OR Ghana OR Guinea OR "Guinea Bissau" OR Kenya OR Lesotho OR Liberia OR Libya OR Madagascar OR Malawi OR Mali OR Mauritania OR Mauritius OR Morocco OR Mozambique OR Namibia OR Niger OR Nigeria OR Rwanda OR "Sao Tome" OR Senegal OR Seychelles OR "Sierra Leone" OR Somalia OR "South Africa" OR Sudan OR "South Sudan" OR Swaziland OR Tanzania OR Togo OR Tunisia OR Uganda OR Zambia OR Zimbabwe) AND AB ("traditional medicine" OR "traditional African medicine" OR "complementary medicine" OR "traditional therapy" OR "complementary therapy" OR "alternative medicine" OR "alternative therapy" OR "natural medicine" OR "herbal medicine" OR herb OR "herbal medicine" OR "medicinal plant" OR "folk medicine" OR "holistic medicine" OR "traditional health practice") AND AB (Matern* OR "maternal health" OR pregnan* OR "pregnancy conditions" OR "maternal care" OR "maternal services" OR mother* OR "child bearing" OR "child birth" OR gestation OR "antenatal care" OR lactat* or "milk secretion" OR "after birth" OR "post birth" OR "post natal care" or labor OR labour OR abortion OR miscarriage OR fertility OR infertility)	61
Medline	AB (Women OR females OR mothers) AND TX (Africa* OR "African migrant" OR "African-born" OR " African immigrant" OR Algeria OR Angola OR Benin OR Botswana OR "Burkina Faso" OR Burundi OR Cameroon OR "Cape Verde" OR "Central African Republic" OR Chad OR Comoros OR "Ivory Coast" OR Congo OR Djibouti OR Egypt OR "Equatorial Guinea" OR Eritrea OR Ethiopia OR Gabon OR Gambia OR Ghana OR Guinea OR "Guinea Bissau" OR Kenya OR Lesotho OR Liberia OR Libya OR Madagascar OR Malawi OR Mali OR Mauritania OR Mauritius OR Morocco OR Mozambique OR Namibia OR Niger OR Nigeria OR Rwanda OR "Sao Tome" OR Senegal OR Seychelles OR "Sierra Leone" OR Somalia OR "South Africa" OR Sudan OR "South Sudan" OR Swaziland OR Tanzania OR Togo OR Tunisia OR Uganda OR Zambia OR Zimbabwe) AND AB ("traditional medicine" OR "traditional African medicine" OR "complementary medicine" OR "traditional therapy" OR "complementary therapy" OR "alternative medicine" OR "alternative therapy" OR "natural medicine" OR "herbal medicine" OR herb OR "herbal medicine" OR "medicinal plant" OR "folk medicine" OR "holistic medicine" OR "traditional health practice") AND AB (Matern* OR "maternal health" OR pregnan* OR "pregnancy conditions" OR "maternal care" OR "maternal services" OR mother* OR "child bearing" OR "child birth" OR gestation OR "antenatal care" OR lactat* or "milk secretion" OR "after birth" OR "post birth" OR "post natal care" or labor OR labour OR abortion OR miscarriage OR fertility OR infertility)	94
ProQuest	ab(Women OR females OR mothers) AND ft(Africa* OR "African migrant" OR "African-born" OR " African immigrant" OR Algeria OR Angola OR Benin OR Botswana OR "Burkina Faso" OR Burundi OR Cameroon OR "Cape Verde" OR "Central African Republic" OR Chad OR Comoros OR "Ivory Coast" OR Congo OR Djibouti OR Egypt OR "Equatorial Guinea" OR Eritrea OR Ethiopia OR Gabon OR Gambia OR Ghana OR Guinea OR "Guinea Bissau" OR Kenya OR Lesotho OR Liberia OR Libya OR Madagascar OR Malawi OR Mali OR Mauritania OR Mauritius OR Morocco OR Mozambique OR Namibia OR Niger OR Nigeria OR Rwanda OR "Sao Tome" OR Senegal OR Seychelles OR "Sierra Leone" OR Somalia OR "South Africa" OR Sudan OR "South Sudan" OR Swaziland OR Tanzania OR Togo OR Tunisia OR Uganda OR Zambia OR Zimbabwe) AND ab("traditional medicine" OR "traditional African medicine" OR "complementary medicine" OR "traditional therapy" OR "complementary therapy" OR "alternative medicine" OR "alternative therapy" OR "natural medicine" OR "herbal medicine" OR herb OR "herbal medicine" OR "medicinal plant" OR "folk medicine" OR "holistic medicine" OR "traditional health practice") AND ab(Matern* OR "maternal health" OR pregnan* OR "pregnancy conditions" OR "maternal care" OR "maternal services" OR mother* OR "child bearing" OR "child birth" OR gestation OR "antenatal care" OR lactat* OR "milk secretion" OR "after birth" OR "post birth" OR "post natal care" OR labor OR labour OR abortion OR miscarriage OR fertility OR infertility)	883
PsycINFO	AB (Women OR females OR mothers) AND TX (Africa* OR "African migrant" OR "African-born" OR " African	17

	immigrant" OR Algeria OR Angola OR Benin OR Botswana OR "Burkina Faso" OR Burundi OR Cameroon OR "Cape Verde" OR "Central African Republic" OR Chad OR Comoros OR "Ivory Coast" OR Congo OR Djibouti OR Egypt OR "Equatorial Guinea" OR Eritrea OR Ethiopia OR Gabon OR Gambia OR Ghana OR Guinea OR "Guinea Bissau" OR Kenya OR Lesotho OR Liberia OR Libya OR Madagascar OR Malawi OR Mali OR Mauritania OR Mauritius OR Morocco OR Mozambique OR Namibia OR Niger OR Nigeria OR Rwanda OR "Sao Tome" OR Senegal OR Seychelles OR "Sierra Leone" OR Somalia OR "South Africa" OR Sudan OR "South Sudan" OR Swaziland OR Tanzania OR Togo OR Tunisia OR Uganda OR Zambia OR Zimbabwe) AND AB ("traditional medicine" OR "traditional African medicine" OR "complementary medicine" OR "traditional therapy" OR "complementary therapy" OR "alternative medicine" OR "alternative therapy" OR "natural medicine" OR "herbal medicine" OR herb OR "herbal medicine" OR "medicinal plant" OR "folk medicine" OR "holistic medicine" OR "traditional health practice") AND AB (Matern* OR "maternal health" OR pregnan* OR "pregnancy conditions" OR "maternal care" OR "maternal services" OR mother* OR "child bearing" OR "child birth" OR gestation OR "antenatal care" OR lactat* or "milk secretion" OR "after birth" OR "post birth" OR "post natal care" or labor OR labour OR abortion OR miscarriage OR fertility OR infertility)	
PubMed	((((Women[Title/Abstract] OR females[Title/Abstract] OR mothers[Title/Abstract])) AND (Africa*[Text Word] OR "African migrant"[Text Word] OR "African-born"[Text Word] OR " African immigrant"[Text Word] OR Algeria[Text Word] OR Angola[Text Word] OR Benin[Text Word] OR Botswana[Text Word] OR "Burkina Faso"[Text Word] OR Burundi[Text Word] OR Cameroon[Text Word] OR "Cape Verde"[Text Word] OR "Central African Republic"[Text Word] OR Chad[Text Word] OR Comoros[Text Word] OR "Ivory Coast"[Text Word] OR Congo[Text Word] OR Djibouti[Text Word] OR Egypt[Text Word] OR "Equatorial Guinea"[Text Word] OR Eritrea[Text Word] OR Ethiopia[Text Word] OR Gabon[Text Word] OR Gambia[Text Word] OR Ghana[Text Word] OR Guinea[Text Word] OR "Guinea Bissau"[Text Word] OR Kenya[Text Word] OR Lesotho[Text Word] OR Liberia[Text Word] OR Libya[Text Word] OR Madagascar[Text Word] OR Malawi[Text Word] OR Mali[Text Word] OR Mauritania[Text Word] OR Mauritius[Text Word] OR Morocco[Text Word] OR Mozambique[Text Word] OR Namibia[Text Word] OR Niger[Text Word] OR Nigeria[Text Word] OR Rwanda[Text Word] OR "Sao Tome"[Text Word] OR Senegal[Text Word] OR Seychelles[Text Word] OR "Sierra Leone"[Text Word] OR Somalia[Text Word] OR "South Africa"[Text Word] OR Sudan[Text Word] OR "South Sudan"[Text Word] OR Swaziland[Text Word] OR Tanzania[Text Word] OR Togo[Text Word] OR Tunisia[Text Word] OR Uganda[Text Word] OR Zambia[Text Word] OR Zimbabwe[Text Word])) AND ("traditional medicine"[Title/Abstract] OR "traditional African medicine"[Title/Abstract] OR "complementary medicine"[Title/Abstract] OR "traditional therapy"[Title/Abstract] OR "complementary therapy"[Title/Abstract] OR "alternative medicine"[Title/Abstract] OR "alternative therapy"[Title/Abstract] OR "natural medicine"[Title/Abstract] OR "herbal medicine"[Title/Abstract] OR herb[Title/Abstract] OR "herbal medicine"[Title/Abstract] OR "medicinal plant"[Title/Abstract] OR "folk medicine"[Title/Abstract] OR "holistic medicine"[Title/Abstract] OR "traditional health practice"[Title/Abstract])) AND (Matern*[Title/Abstract] OR "maternal health"[Title/Abstract] OR pregnan*[Title/Abstract] OR "pregnancy conditions"[Title/Abstract] OR "maternal care"[Title/Abstract] OR "maternal services"[Title/Abstract] OR mother*[Title/Abstract] OR "child bearing"[Title/Abstract] OR "child birth"[Title/Abstract] OR gestation[Title/Abstract] OR "antenatal care"[Title/Abstract] OR lactat*[Title/Abstract] OR "milk secretion"[Title/Abstract] OR "after birth"[Title/Abstract] OR "post birth"[Title/Abstract] OR "post natal care"[Title/Abstract] OR labor[Title/Abstract] OR labour[Title/Abstract] OR abortion[Title/Abstract] OR miscarriage[Title/Abstract] OR fertility[Title/Abstract] OR infertility[Title/Abstract])	112
SCOPUS	ABS(Women OR females OR mothers) AND ABS(Africa* OR "African migrant" OR "African-born" OR " African immigrant" OR Algeria OR Angola OR Benin OR Botswana OR "Burkina Faso" OR Burundi OR Cameroon OR "Cape Verde" OR "Central African Republic" OR Chad OR Comoros OR "Ivory Coast" OR Congo OR Djibouti OR Egypt OR "Equatorial Guinea" OR Eritrea OR Ethiopia OR Gabon OR Gambia OR Ghana OR Guinea OR "Guinea Bissau" OR Kenya OR Lesotho OR Liberia OR Libya OR Madagascar OR Malawi OR Mali OR Mauritania OR Mauritius OR Morocco OR Mozambique OR Namibia OR Niger OR Nigeria OR Rwanda OR "Sao Tome" OR Senegal OR Seychelles OR "Sierra Leone" OR Somalia OR "South Africa" OR Sudan OR "South Sudan" OR Swaziland OR Tanzania OR Togo OR Tunisia OR Uganda OR Zambia OR Zimbabwe) AND ABS("Traditional medicine" OR "traditional African medicine" OR "complementary medicine" OR "traditional therapy" OR "complementary therapy" OR "alternative medicine" OR "alternative therapy" OR "natural medicine" OR	248

	herb OR "herbal medicine" OR "medicinal plant" OR "folk medicine" OR "holistic medicine" OR "traditional health practice") AND ABS(Matern* OR "maternal health" OR pregnan* OR "pregnancy conditions" OR "maternal care" OR "maternal services" OR mother* OR "child bearing" OR "child birth" OR gestation OR "antenatal care" OR lactat* or "milk secretion" OR "after birth" OR "post birth" OR "post natal care" or labor OR labour OR abortion OR miscarriage OR fertility OR infertility)	
Embase	(Women or woman or females).ab. and (((((((((((((((((((((((((((((((African-born or African) adj migrant) or African) adj immigrant) or Africa* or Sub-Saharan) adj Africa) or North) adj Africa) or West) adj Africa) or Algeria* or Angola* or Benin* or Botswana* or Burkina) adj Faso) or Burundi* or Cameroon* or Cape) adj Verde) or Central) adj African adj Republic) or Chad* or Comoros* or Ivory) adj Coast) or Congo* or Djibouti* or Egypt* or Equatorial) adj Guinea) or Eritrea* or Ethiopia* or Gabon* or Gambia* or Ghana* or Guinea* or Guinea) adj Bissau) or Kenya* or Lesotho* or Liberia* or Libya* or Madagascar* or Malawi* or Mali* or Mauritania* or Mauritius* or Morocco* or Mozambique* or Namibia* or Niger* or Nigeria* or Rwanda* or Sao) adj Tome) or Senegal* or Seychelles* or Sierra) adj Leone) or Somalia* or South) adj Africa) or Sudan* or South) adj Sudan) or Swaziland* or Tanzania* or Togo* or Tunisia* or Uganda* or Zambia* or Zimbabwe*).tw. and (Traditional medicine or traditional African medicine or complementary medicine or traditional therapy or complementary therapy or alternative medicine or alternative therapy or natural medicine or herbal medicine or herb* or folk medicine or holistic medicine or traditional health practice).tw. and ((((((((((((((((((((Matern* or maternal) adj health) or pregnan* or pregnancy) adj conditions) or maternal) adj care) or maternal) adj services) or mother* or child) adj bearing) or child) adj birth) or gestation or antenatal) adj care) or lactat* or milk) adj secretion) or after) adj birth) or post) adj birth) or postnatal) adj care) or labor or labour or abortion or miscarriage or fertility or infertility).ab.	25
Infomit	(AB:Women OR AB:woman OR AB:females) AND (AB:“African-born” OR AB:“African AB:migrant” OR AB:“African AB:immigrant” OR AB:Africa* OR AB:“Sub-Saharan AB:Africa” OR AB:“North AB:Africa” OR AB:“West AB:Africa” OR AB:Algeria* OR AB:Angola* OR AB:Benin* OR AB:Botswana* OR AB:“Burkina AB:Faso” OR AB:Burundi* OR AB:Cameroon* OR AB:“Cape AB:Verde” OR AB:“Central AB:African AB:Republic” OR AB:Chad* OR AB:Comoros* OR AB:“Ivory AB:Coast” OR AB:Congo* OR AB:Djibouti* OR AB:Egypt* OR AB:“Equatorial AB:Guinea” OR AB:Eritrea* OR AB:Ethiopia* OR AB:Gabon* OR AB:Gambia* OR AB:Ghana* OR AB:Guinea* OR AB:“Guinea AB:Bissau” OR AB:Kenya* OR AB:Lesotho* OR AB:Liberia* OR AB:Libya* OR AB:Madagascar* OR AB:Malawi* OR AB:Mali* OR AB:Mauritania* OR AB:Mauritius* OR AB:Morocco* OR AB:Mozambique* OR AB:Namibia* OR AB:Niger* OR AB:Nigeria* OR AB:Rwanda* OR AB:“Sao AB:Tome” OR AB:Senegal* OR AB:Seychelles* OR AB:“Sierra AB:Leone” OR AB:Somalia* OR AB:“South AB:Africa” OR AB:Sudan* OR AB:“South AB:Sudan” OR AB:Swaziland* OR AB:Tanzania* OR AB:Togo* OR AB:Tunisia* OR AB:Uganda* OR AB:Zambia* OR AB:Zimbabwe*) AND (AB:“Traditional AB:medicine” OR AB:“traditional AB:African AB:medicine” OR AB:“complementary AB:medicine” OR AB:“traditional AB:therapy” OR AB:“complementary AB:therapy” OR AB:“alternative AB:medicine” OR AB:“alternative AB:therapy” OR AB:“natural AB:medicine” OR AB:“herbal AB:medicine” OR AB:herb* OR AB:“folk AB:medicine” OR AB:“holistic AB:medicine” AB:“traditional AB:health AB:practice”;) AND (AB:Matern* OR AB:“maternal AB:health” OR AB:pregnan* OR AB:“pregnancy AB:conditions” OR AB:“maternal AB:care” OR AB:“maternal AB:services” OR AB:mother* OR AB:“child AB:bearing” OR AB:“child AB:birth” OR AB:gestation OR AB:“antenatal AB:care” OR AB:lactat* AB:or AB:“milk AB:secretion” OR AB:“after AB:birth” OR AB:“post AB:birth” OR AB:“post AB:natal AB:care” AB:or AB:labor OR AB:labour OR AB:abortion OR AB:miscarriage OR AB:fertility OR AB:infertility)	509
